# Principles of Surgery

**Published:** 1891-10

**Authors:** 


PRINCIPLES OF SURGERY. By N. Senn, M. D., Ph. D., Milwaukee, Wis., Professor of Principles of Surgery and Surgical Pathology in Rush Medical College, Chicago, Ill., etc. Illustrated with 109 wood engravings. Philadelphia and

London. F. A. Davis, Publisher. 1890. Price, Cloth, $4.50 ; Sheep, $5.50, net.

The aim of this work is to bring up to date the recent investigations in pathology, in its bearings upon surgical treatment. It is, in fact, a work on pathology, and as such, finds its place in the voluminous literature on the subject. Its chief use, as the author states in his preface, is to bring before the student and practitioner the results of pathological investigation in a convenient form. S. T.

